# Ocular Surface Inflammation as a Driver of Cornea Limbal Stem Cell Deficiency: Mechanisms and Implications

**DOI:** 10.3390/ijms27114718

**Published:** 2026-05-23

**Authors:** Yura Choi, Mi-Young Jung, Eunsun Han, Choul Yong Park

**Affiliations:** Department of Ophthalmology, Samsung Medical Center, Sungkyunkwan University School of Medicine, Seoul 06351, Republic of Korea; ychoi.op@gmail.com (Y.C.); myjung202@gmail.com (M.-Y.J.);

**Keywords:** eye, limbus, stem cell, inflammation, limbal stem cell deficiency, treatment

## Abstract

Ocular surface inflammation is a major disruptor of corneal epithelial homeostasis and a key driver of limbal stem cell deficiency (LSCD). Limbal stem cells (LSCs), residing within the specialized limbal niche, maintain corneal transparency through continuous epithelial renewal and by preventing conjunctival encroachment onto the corneal surface. Chronic or severe inflammatory insults—stemming from systemic autoimmune disorders, ocular surface diseases, infections, trauma, or environmental stressors—can damage both LSCs and their microenvironment, ultimately leading to limbal insufficiency. This review synthesizes current insights into the mechanisms by which inflammation impairs LSC survival, including cytokine-mediated cytotoxicity, oxidative stress, immune cell infiltration, and disruption of essential signaling pathways such as Wnt, Notch, and BMP. The distinction between LSC depletion and LSC dysfunction is highlighted, as residual stem cells may persist even in clinically advanced disease and can regenerate the corneal surface once the inflammatory milieu is corrected. Clinical manifestations, staging systems, and diagnostic markers—including p63α, ABCG2, and additional emerging molecular indicators—are summarized to support accurate assessment of LSCD severity. Current therapeutic strategies, ranging from anti-inflammatory medical management to surgical approaches such as SLET, CLET, and allogeneic transplantation, are reviewed alongside evolving regenerative and cell-based therapies. By integrating mechanistic understanding with clinical implications, this review underscores the critical interplay between inflammation and limbal niche failure and emphasizes the importance of early recognition and targeted intervention to preserve or restore LSC function.

## 1. Introduction

The ocular surface, a vital component of the visual system, comprises the cornea, conjunctiva, and the tear film [1]. This intricate structure is essential for maintaining clear vision, with the cornea playing a critical role in refracting light onto the retina [1]. The transparency and structural integrity of the cornea are perpetually maintained by a sophisticated process of epithelial renewal, a function primarily attributed to limbal stem cells (LSCs) [2,3]. These specialized cells reside in the limbus, a narrow transitional zone located at the junction between the cornea and the conjunctiva [2,4]. This region serves as a reservoir of progenitor cells that continuously replenish the corneal epithelium [4].

Ocular surface inflammation refers to a wide range of conditions that disrupt the delicate equilibrium of the ocular surface, impacting its function and health [5]. These inflammatory processes can arise from multiple factors, ranging from systemic diseases to localized injuries [5,6]. When ocular surface inflammation becomes severe or chronic, it disrupts the ocular surface’s homeostasis and, more critically, damages LSCs and their supportive microenvironment, the limbal niche [4,7]. Such damage can lead to a debilitating condition known as limbal insufficiency or limbal stem cell deficiency (LSCD), potentially resulting in vision loss and ocular discomfort [3,4].

This review aims to provide a comprehensive exploration of the intricate relationship that exists between ocular surface inflammation and the subsequent development of LSCD. By examining the definitions, causes, and underlying mechanisms of both conditions, this report will elucidate the pathological pathways through which inflammation can lead to LSCD. Furthermore, it will detail the clinical signs and symptoms that indicate limbal insufficiency as consequences of ocular surface inflammation, summarize current treatment strategies, and highlight recent advancements and emerging therapeutic approaches in this critical area of ophthalmic research and clinical practice.

## 2. Understanding Ocular Surface Inflammation

Ocular surface inflammatory disorders comprise a broad spectrum of conditions marked by chronic inflammation that disrupts key ocular tissues, particularly the cornea and conjunctiva [8,9,10]. Their etiologies are diverse and frequently linked to systemic diseases—such as graft-versus-host disease (GVHD), mucous membrane pemphigoid (MMP), Sjögren’s syndrome (SS), and allergic disorders—that drive persistent inflammatory responses on the ocular surface [8,10,11,12,13] (Table 1). In addition to systemic contributors, several local factors—including infections, mechanical irritation, trauma, and meibomian gland dysfunction (MGD)—can also precipitate or exacerbate ocular surface damage [14]. Tear film instability resulting from MGD can compromise the ocular epithelium and induce secondary inflammation, while primary ocular surface inflammation may further impair meibomian gland function, creating a bidirectional cycle of disease progression [14]. Dry eye syndrome is a prevalent, multifactorial disorder of the ocular surface characterized by a pronounced inflammatory component that often sustains and amplifies itself in a vicious cycle [9,14]. A range of other ocular surface diseases—including blepharitis, neurotrophic keratitis, ocular rosacea, and allergic eye disease—are also well-recognized drivers of persistent ocular inflammation [8,15]. Beyond disease-related factors, numerous external and systemic influences such as prolonged contact lens wear, certain therapeutic medications (e.g., antihistamines, antidepressants, and preserved glaucoma treatments), environmental stressors (dry air, wind, smoke), aging, and hormonal fluctuations can further initiate or exacerbate inflammatory processes on the ocular surface [8,16,17,18,19,20].

## 3. Understanding Limbal Stem Cells and Limbal Stem Cell Deficiency

### 3.1. Role of Limbal Stem Cells (LSCs)

**Figure 1:**
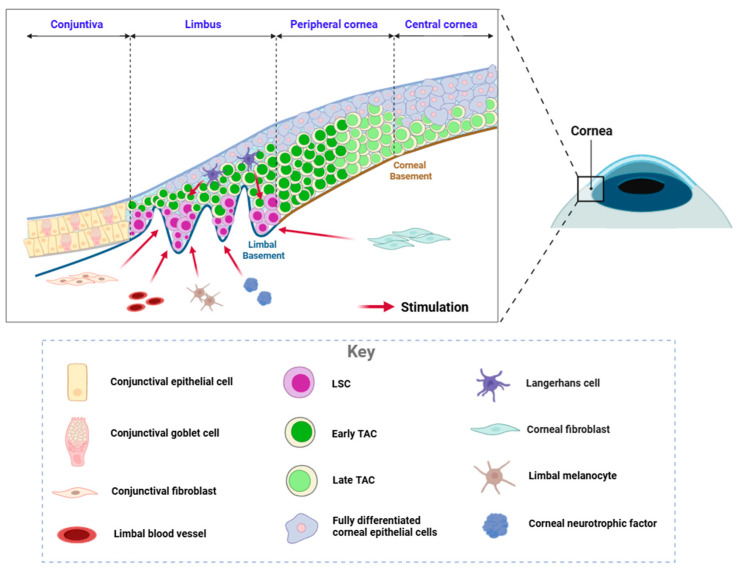
Schematic illustration of the ocular surface showing the transition from conjunctiva to cornea through the limbus. Limbal stem cells (LSCs) differentiate into transient amplifying cells (TACs) and subsequently into mature corneal epithelial layers. The limbal niche, where LSCs reside, is supported by fibroblasts, melanocytes, Langerhans cells, and neurotrophic elements distributed along the basement membranes. Their coordinated interplay maintains LSC homeostasis and regulates the supply of TACs. Created in BioRender. Jung, M. (2026) https://BioRender.com/5kod2u1 (accessed on 15 March 2026).

LSCs, with their unique characteristics like slow turnover and high proliferative potential, are located in the limbus within structures known as limbal crypt [21,22,23] (Figure 1). The primary function of LSCs is to ensure the continuous renewal of the corneal epithelium, which is essential for maintaining corneal transparency and overall ocular surface health [22]. This process involves the LSCs undergoing cell division to produce daughter cells that then differentiate into transit amplifying cells (TACs) [21,22]. Although several animal studies have suggested that some central corneal epithelial cells, rather than LSCs, possess intrinsic self-renewal properties and the potential to migrate to limbus, more definitive evidence is still required in humans [24]. The TACs are capable of rapid proliferation and subsequently mature into the fully differentiated corneal epithelial cells that migrate centripetally across the cornea, following the widely accepted XYZ hypothesis of corneal epithelial maintenance [21,23]. Crucially, LSCs also prevent the migration of conjunctival epithelial cells onto the corneal surface by forming a direct mechanical barrier and by supporting the limbal niche microenvironment [22]. This barrier function is essential for preserving the cornea’s avascularity and its highly specialized epithelial phenotype, both of which are required for maintaining clear vision [25]. When LSCs are damaged or their numbers are reduced, the corneal epithelium cannot effectively repair itself, leading to the various signs and symptoms of LSCD [12]. LSCD can manifest in varying degrees, from partial deficiency, which is characterized by focal disruption of limbus and limited conjunctival ingrowth, to total deficiency, where the entire cornea is affected by vascularized conjunctiva [12,26]. It is also important to distinguish between a true deficiency in the number of LSCs and a functional impairment in which the cells are present but unable to perform their normal regenerative roles, as this distinction has significant implications for selecting appropriate treatment strategies.

### 3.2. Markers of Limbal Stem Cells of the Cornea

Identifying reliable markers for LSCs is essential not only for advancing our understanding of their biology but also for improving diagnostic accuracy and developing targeted therapies for LSCD, a condition that can ultimately lead to severe vision impairment or blindness. Although numerous candidate markers have been proposed, each reflects only a subset of LSC characteristics, and none has yet demonstrated the specificity and consistency required to serve as a definitive, stand-alone marker. This ongoing challenge underscores the complexity of LSC biology and highlights the need for a combinatorial marker approach, integrating molecular, functional, and spatial characteristics to more accurately distinguish LSCs from neighboring epithelial populations [14,27,28] (Table 2).

One of the most extensively studied markers of LSCs is the *p63* gene, particularly the ΔNp63α isoform [14,27,28,29]. This transcription factor is localized to the nuclei of limbal basal epithelial cells and is essential for maintaining their proliferative and regenerative capacity [30,31]. However, p63 is not entirely specific to LSCs, as it is also present in other basal epithelial cell populations, limiting its utility as a definitive standalone marker [32].

ABCG2, an ATP-binding cassette transporter, is another important LSC-associated marker [33]. It is predominantly expressed in the basal limbal epithelium and correlates with stem cell characteristics, including enhanced colony-forming efficiency. ABCG2 is therefore considered a promising candidate for isolating and identifying LSCs. Similarly, ABCB5—another ATP-binding cassette transporter—has been implicated in corneal epithelial development and repair and is proposed as an additional LSC marker [34,35].

Several cytoskeletal and adhesion-related proteins also contribute to defining the limbal epithelial phenotype. Keratin 15 (K15) is expressed in the basal cells of the limbal and conjunctival epithelium, but not in the central cornea, making it useful for distinguishing limbal epithelium from other ocular surface tissues [36]. N-cadherin, a cell–cell adhesion molecule, is expressed by putative LSCs and melanocytes within the limbal niche and is thought to help maintain the progenitor state of limbal epithelial cells. Vimentin, an intermediate filament protein, is likewise present in limbal basal cells and contributes to cytoskeletal organization and cell adhesion [27].

Transcription factors also play a pivotal role in defining LSC identity. PAX6, a master regulator of ocular development, is expressed in corneal epithelial progenitors and influences limbal epithelial cell fate decisions [37]. Although not exclusive to LSCs, its significance is highlighted by the high incidence of LSCD in individuals with congenital aniridia caused by *PAX6* mutations, underscoring its essential role in limbal embryogenesis [38]. More recently, Sox9 has been identified as a transcription factor preferentially expressed in LSCs and shown to be critical for regulating asymmetric cell division within the corneal epithelium [39].

Advances in single-cell RNA sequencing have further expanded the repertoire of putative LSC markers. GPHA2 (glycoprotein hormone subunit alpha 2) is highly expressed in cells within the limbal crypts, supporting its relevance to the LSC niche [40]. Additional molecules—including apolipoprotein E, p75NTR, tetraspanin-1, interferon-induced transmembrane protein 3 (IFITM3), and activating transcription factor 3 (ATF3)—have also been proposed as potential LSC markers [41].

While each of these markers provides valuable insight into LSC identity and function, no single marker offers absolute specificity. Consequently, the most effective current strategy relies on using a combination of markers to identify and characterize LSC populations. For example, the co-expression of ΔNp63α and ABCG2 is frequently employed to enrich for cells with LSC-like properties [23,27,29,42,43].

The clinical significance of these markers lies in their potential to aid in the diagnosis of LSCD through techniques like impression cytology and in vivo confocal microscopy. Furthermore, identifying and isolating LSCs using these markers is crucial for the development of cell-based therapies, such as cultivated limbal epithelial transplantation (CLET) aimed at restoring the corneal surface in patients with LSCD [43].

**Table 2:** Representative Markers of Limbal Stem Cells and Their Characteristics.

Marker	Expression Pattern	Functional Relevance	Limitation/Notes	Reference
ΔNp63α(p63)	Nuclei of limbal basal cells	Maintains proliferative potential	Not fully specific to LSCs	[14]
ABCG2	Limbal basal cells	Associated with colony-forming efficiency	Also expressed in other stem-like cells	[33]
ABCB5	Limbal epithelium	Corneal epithelial development and repair	Proposed LSC marker	[34]
K15	Basal cells of limbus & conjunctiva	Helps distinguish limbal phenotype	Absent in central cornea	[36]
N-Cadherin	Putative LSCs, melanocytes	Maintains progenitor phenotype	Niche-associated marker	[27]
Pax6	Corneal epithelial lineage	Lineage specification; aniridia-associated LSCD	Not LSC-specific	[38]
Sox9	Limbal stem/progenitor cells	Regulates asymmetric cell fate	Emerging marker	[39]
GPHA2	Limbal crypt cells	Enriched in quiescent LSC population	Recently identified	[40]

### 3.3. Transit Amplifying Cells (Tac)

Corneal epithelial TACs are highly proliferative progenitors that function as the intermediate population between quiescent LSCs and terminally differentiated corneal epithelial cells [44]. Molecularly, TACs are distinguished from LSCs by their strong upregulation of proliferation-associated genes and the early activation of corneal-specific differentiation programs. In one study, single-cell transcriptomic analyses identified TACs as a distinct cluster—approximately 3.2% of limbal cells—enriched with markers such as MKI67, TOP2A, CCNB1, BIRC5, FOXM1, and PLK1 [45]. Functionally, TACs are the most mitotically active population in the corneal epithelium and serve as the primary drivers of tissue renewal.

Under homeostatic conditions, they exhibit a relatively long cell-cycle duration of about 72 h and typically undergo at least two rounds of division before moving centripetally. Overall, the full turnover of the corneal epithelium—from basal proliferation to superficial desquamation—occurs over an estimated 7 to 14 days [46]. Transcriptomic profiling shows that nearly 98% of TACs reside in the S or G2/M phases, in contrast to LSCs, which remain largely in G0/G1 [45]. Following injury, TACs markedly accelerate their cycling to support rapid re-epithelialization, with regeneration rates reaching over 60 μm per hour in animal models [47]. Various pathological cues stimulate TAC proliferation after injury (Table 3).

### 3.4. Factors Maintaining Limbal Stem Cell Survival

The survival and function of LSCs depend on the limbal stem cell niche, a specialized microenvironment at the limbus [56,57] (Figure 1). This niche consists of cellular components—limbal stromal cells (including mesenchymal stem cells and niche cells), melanocytes, immune cells (Langerhans cells, T-lymphocytes), and vascular and nerve cells—as well as non-cellular elements like the extracellular matrix and basement membrane, which provide structural and regulatory support [56,57,58,59,60] (Table 4). Key anatomical structures, such as the palisades of Vogt and limbal crypts, help protect LSCs [56,57,58,59,60,61,62].

LSCs maintenance involves complex interactions. Limbal niche supplies essential support via its cellular and non-cellular components, including extracellular matrix (ECM) such as hyaluronan and basement membrane. Furthermore, growth factors like EGF, NGF, HGF, insulin like growth factor-1 (IGF-1), and pigment epithelium derived factor (PEDF) promote LSCs proliferation, differentiation, and survival [63,64]. Vascular nutrition and neuronal regulation are also vital [56,65].

**Figure 2:**
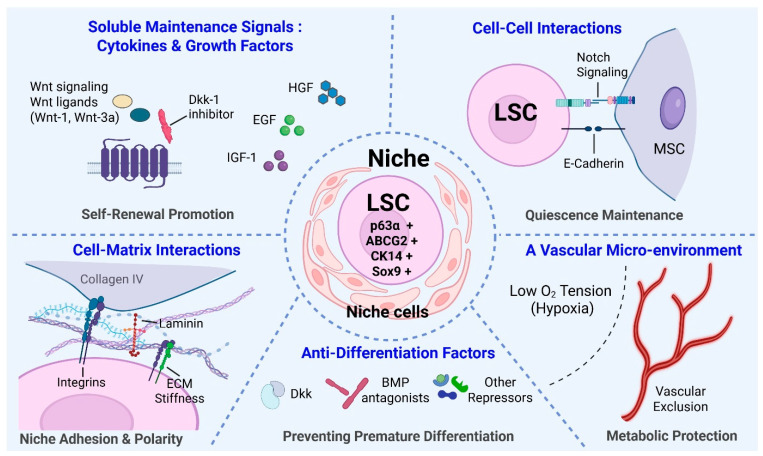
Regulation to maintain limbal stem cell stemness and survival. Soluble factors such as Wnt ligands, HGF, EGF, and IGF-1 promote LSC self-renewal, whereas inhibitors including Dkk-1 and BMP antagonists suppress premature differentiation. Direct cell–cell interactions between LSC and MSC, including Notch signaling and E-cadherin contacts, help maintain LSC quiescence. Interactions with extracellular matrix components such as collagen IV, laminin, and integrins support adhesion and polarity of LSC. Avascular and hypoxic microenvironment provides metabolic protection of LSC. Together, these coordinated cues preserve LSC identity and sustain long-term regenerative function in limbal niche. Created in BioRender. Jung, M. (2026) https://BioRender.com/wvd9a8d (accessed on 15 March 2026).

At the molecular level, LSC fate is governed by finely tuned signaling networks. Among these, Wnt, Notch, and BMP pathways play central roles in regulating LSC behavior (Figure 2). Wnt signaling supports LSC proliferation and maintains their undifferentiated state, thereby supporting self-renewal [66]. Notch signaling coordinates proliferation and differentiation, guiding the transition from LSCs to transient amplifying cells and ultimately to mature corneal epithelial cells. In contrast, BMP signaling maintains LSC quiescence and prevents excessive differentiation, helping preserve niche stability [67]. Additional pathways—including Shh, YAP, and TGF-β—and transcription factors such as p63 and Sox9 further contribute to LSC regulation. Recent proteomic analyses have shown that LSC-derived exosomes contain key signaling mediators, including S100A2, KRT17, collagen type 17A1, and multiple growth factors, underscoring that LSCs not only respond to their microenvironment but also actively shape it [68].

Neurotrophic support is another essential component of LSC maintenance. LSCD frequently coexists with neurotrophic keratitis [69]. Supplementation of LSC cultures with NGF prolongs LSC lifespan and increases the expression of ΔNp63α and ABCG2, while both NGF and its receptor p75NTR are expressed in early LSC cultures and downregulated during differentiation [70]. Ciliary neurotrophic factor (CNTF) enhances colony-forming efficiency and stimulates LSC proliferation and migration [71]. Clinically, cenegermin, a recombinant human NGF, has successfully restored the corneal epithelium in patients with combined LSCD and neurotrophic keratitis refractory to other treatment [72].

The limbal basement membrane itself provides a highly specialized microenvironment for LSCs. It contains laminin α1, α2, and β1 chains, laminin γ3, agrin, BM40/SPARC (Secreted Protein Acidic and Rich in Cysteine), and tenascin-C [73,74]. These components co-localize with clusters of cells expressing ABCG2, p63, and K19—but lacking K3, Cx43, desmoglein, and integrin α2—indicating the presence of putative stem and early progenitor cells within the basal epithelium of the limbal palisades [75,76,77]. SPARC further enhances stemness by activating JNK and p38-MAPK signaling pathways, increasing the expression of markers such as ABCG2, Bmi-1, and Ki67 [78].

### 3.5. Characteristics of Limbal Stem Cell Deficiency

A hallmark characteristic of LSCD is conjunctivalization of corneal surface, a process where the normal corneal epithelial cells are replaced by conjunctival epithelial cells often identified by the presence of goblet cells on the corneal surface [26,79]. Other characteristics include corneal neovascularization, where new blood vessels grow into the normally avascular cornea, and the persistent epithelial defects which means abnormal regeneration of cornea epithelium [2,26]. Upon clinical examination using a slit lamp, the corneal surface loses its characteristic smoothness and transparency and may exhibit a dull and irregular light reflex. A characteristic pattern known as a “whorled” epithelium is often observed, along with the absence or attenuation of the normal limbal palisades of Vogt [26,79].

When the eye is stained with fluorescein, a stippled pattern may appear on the corneal surface, indicating the encroachment of conjunctival epithelium [79]. In more advanced stages of limbal insufficiency, several other signs may become evident, including a haze within the corneal epithelium, corneal neovascularization, scarring of the corneal stroma, and conjunctival hyperemia [26,80].

Patients may also develop persistent epithelial defects, which can sometimes progress to corneal ulceration, melting, or even perforation [26]. The formation of a fibrovascular pannus, keratinization and calcification on the ocular surface are also possible findings [2,80]. LSCD is often accompanied by abnormalities of the corneal nerves [69]. Reductions in total sub-basal nerve density and long nerve density were observed by increases in nerve tortuosity in LSCD [65]. Even complete drop out of sub-basal nerve plexus was observed in half of late LSCD [65]. Therefore, patients frequently report significantly reduced corneal pain perception relative to the severity of their ocular surface findings.

From a symptomatic standpoint, patients in the early stages typically experience eye irritation, tearing, pain, photophobia, and intolerance to contact lenses. As the disease progresses, reduced visual acuity becomes a prominent complaint due to the replacement of transparent corneal epithelium with less transparent conjunctival tissue.

### 3.6. Classification and Staging of Limbal Stem Cell Deficiency

Although LSCD has historically been described using simplified classifications such as partial versus total deficiency, such binary approaches do not adequately reflect the heterogeneity of the disease. In particular, these traditional classifications fail to account for differences in underlying etiology, the extent of limbal involvement, and involvement of the central visual axis, all of which have important implications for treatment selection and prognosis.

LSCD can be broadly classified into genetic or acquired forms by the etiology [10]. Acquired LSCD is more common and can result from various factors, including chemical or thermal injuries, contact lens wear, ocular surgery, radiation exposure, and inflammatory conditions. Genetic causes often include conditions like aniridia. Notably, LSCD can be reversible, particularly in its early stages, with appropriate management focused on addressing underlying limbal niche dysfunction where inflammation often plays a significant role. The severity of LSCD is often staged based on the extent of corneal epithelial damage and limbal involvement, as defined by the International LSCD Working Group [80].

**Figure 3:**
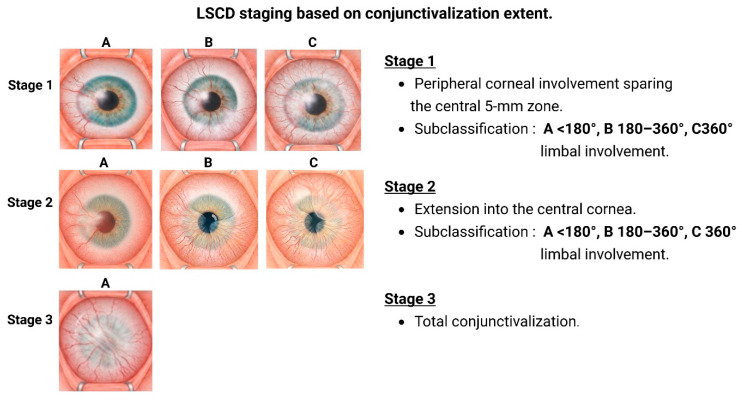
Staging of limbal stem cell deficiency (LSCD). Schematic representation of LSCD staging based on the extent of corneal conjunctivalization. Stage 1 shows peripheral corneal involvement sparing the central 5 mm zone, with limbal involvement classified as <180° (**A**), 180–360° (**B**), or 360° (**C**). Stage 2 demonstrates extension into the central cornea with the same subclassification. Stage 3 represents total corneal conjunctivalization, indicating complete loss of limbal stem cell function. Created in BioRender. Jung, M. (2026) https://BioRender.com/b24amen (accessed on 10 March 2026).

To address these limitations, the International LSCD Working Group proposed a standardized classification and staging system based on objective and clinically reproducible parameters (Figure 3). This system integrates etiologic classification with anatomical assessment of limbal involvement and corneal epithelial integrity, thereby providing a clinically meaningful framework for stratifying disease severity and guiding therapeutic decision-making.

The main stages of LSCD, as proposed by International LSCD Working Group, are based on the health of the corneal epithelium, particularly within the central 5 mm area, which is critical for visual acuity, and are classified as Stages 1 through 3 [80]. Disease severity is further subclassified according to the circumferential extent of limbal involvement, which is categorized as stage A, stage B, or stage C (Table 5).

Because the extent of remaining limbal stem cells strongly influences prognosis and treatment strategy, each stage is further subdivided by the circumferential degree of limbal involvement. Stage A involves less than 50% of the limbus and may be suitable for procedures like SLET, where a small biopsy from the healthy eye can restore the deficient area. Stage B involves 50–100% of the limbus, indicating a more substantial loss of stem cells and often requiring 360-degree keratolimbal allograft (CLAL) or cultivated limbal epithelial transplantation (CLET) to expand the available cells. Stage C represents complete (100%) limbal involvement. In Stage 1C, the central cornea may still appear normal, but the absence of a functional limbal barrier places the eye at high risk for future conjunctivalization, warranting close monitoring and possibly early surgical intervention.

## 4. Differentiation Between LSC Depletion and LSC Dysfunction

Distinguishing between limbal stem cell (LSC) depletion and LSC dysfunction is critical for guiding appropriate therapeutic strategies (Table 6). In cases of true LSC depletion, the primary goal is to restore the lost stem cell population through transplantation or regenerative approaches [22]. Conversely, when LSC dysfunction is present, treatment should focus on addressing the underlying factors—such as inflammation, genetic defects, or environmental stressors—that impair stem cell function, thereby enabling the remaining LSCs to recover and resume their physiological roles [81,82]. However, it should be considered that many patients are likely to have both LSC depletion and dysfunction simultaneously. Considering that patients have been reported to recover a normal corneal surface even after chemical injury destroying about 75% of the limbus, it implies that limbal stem cells have the potential to supply nearly normal corneal epithelium with only about 25% remaining [83]. Interestingly, recent evidence indicates that patients with ocular surface chemical burns who appear to have total LSCD on clinical examination may still retain areas of normal limbal epithelium within the deep limbal region [84].

Clinically, both LSC dysfunction and depletion may coexist in the early stages of disease [22]. However, without timely intervention, LSC dysfunction often progresses to irreversible depletion. Therefore, early diagnosis and management of LSC dysfunction is essential to prevent the transition to complete stem cell loss. Interestingly, studies comparing the origin of corneal epithelial cells following clinical improvement in eyes treated with limbal allografts have shown that, in a significant number of cases, donor-derived DNA is absent, while corneal epithelial cells expressing the recipient’s DNA are present [81,82,85]. This finding suggests that even in relatively advanced stages of LSCD, a subset of recipient LSCs may survive. Once the ocular surface environment is restored, these residual stem cells appear capable of regenerating the corneal epithelium [86].

Severe acute insults—such as chemical burns, radiation exposure, or surgical trauma—are typically associated with true LSC depletion. In contrast, conditions like aniridia and chronic ocular surface inflammation tend to present initially with a preserved stem cell population but impaired function [86]. In cases of total LSC depletion, patients exhibit extensive 360-degree conjunctivalization, where the entire corneal surface, including the central region, is replaced by conjunctival epithelial cells [79]. This is reflected in impression cytology by the absence of corneal epithelial markers such as keratin 3 or keratin 12 (CK12) and positive Muc5ac mucin stain for identifying goblet cells [32,79]. On the other hand, LSC dysfunction is characterized by patchy or peripheral conjunctivalization, with CK12 expression still detectable in the central cornea—indicating the presence of functional corneal epithelial cells [79].

## 5. The Connection Between Inflammation and Stem Cell Damage

Under normal physiological conditions, tissue stem cells maintain tissue homeostasis by dividing at a regulated pace and continuously supplying new cellular components. However, in an inflammatory environment, tissue damage increases, placing a greater burden on stem cells to accelerate repair and regeneration. Stem cell activity is governed by intrinsic regulatory mechanisms as well as extrinsic signals derived from the surrounding niche, among which inflammation is a key factor. Numerous studies have shown that inflammatory conditions can impair tissue repair across multiple organ systems [87].

In addition, during acute inflammation, tissue stem cells rapidly shift into a highly proliferative state to replenish the cell population. However, under chronic inflammatory conditions, this regenerative mechanism becomes exhausted, leading to an overall decline in stem cell function [88]. Therefore, the effects of inflammation on tissue stem cells differ between acute and chronic conditions. The relationship between inflammation and aging is well recognized. This line of investigation is largely driven by the fact that a state of chronic low-grade inflammation, known as inflammaging, exists in the elderly and is considered a likely mediator of various age-associated phenotypes [89]. According to this concept, chronic inflammation impairs the body’s overall regenerative capacity and accelerates the aging process [90].

**Figure 4:**
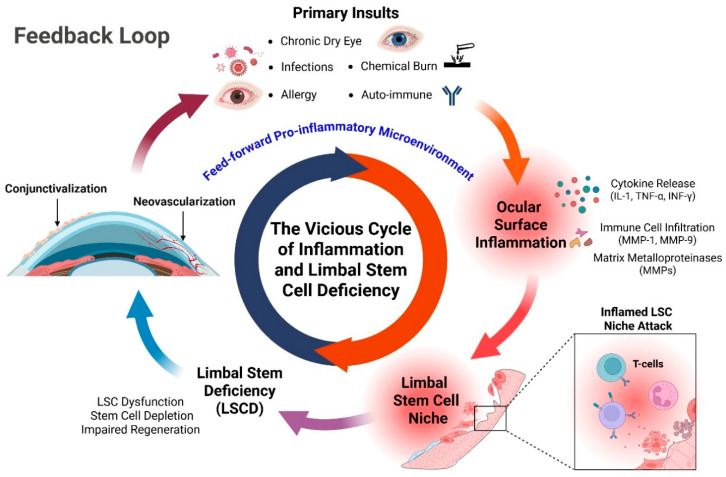
Schematic representation of the vicious cycle between ocular surface inflammation and limbal stem cell deficiency (LSCD). Primary insults—including dry eye, infection, chemical injury, allergy, and autoimmune disease—initiate inflammatory responses characterized by cytokine release, immune cell infiltration, and matrix metalloproteinase activity. This inflammatory milieu damages the limbal stem cell niche, leading to LSC dysfunction, depletion, and impaired epithelial regeneration. Resulting conjunctivalization and neovascularization further amplify inflammation, creating a self-perpetuating feed-forward cycle. Created in BioRender. Jung, M. (2026) https://BioRender.com/2ksh80v (accessed on 23 March 2026).

Multiple mechanisms have been implicated in inflammation-induced stem cell damage (Figure 4). Persistent inflammatory insults can disrupt the stem cell niche, leading to stem cell aging and death. In addition, alterations in cytokine profiles during inflammation, along with interactions between stem cells and various inflammatory cells recruited to the site, can further impair normal stem cell function [87]. For example, TNFR1-deficient mice exhibited markedly increased cell proliferation in the dentate gyrus and a higher number of newborn neurons in the granule cell layer, suggesting that the pro-inflammatory cytokine TNF-α suppresses neural progenitor stem cell activity [91]. Similarly, in a corneal alkali burn mouse model, systemic administration of infliximab, a TNF-α inhibitor, significantly improved LSC survival and reduced corneal scarring [92].

Although the effects of acute and chronic ocular surface inflammation on LSCs have not yet been thoroughly investigated, insights can be drawn from studies on other tissue stem cells. Chen et al. investigated the mechanisms underlying olfactory sensory nerve (OSN) loss using a mouse genetic model of inducible olfactory inflammation that recapitulates the progression of inflammation and OSN degeneration observed in patients with chronic rhinosinusitis [93]. They reported that inflammation initially stimulates horizontal basal stem cells to proliferate and generate new OSNs, whereas chronic inflammation ultimately suppresses OSN production [93]. Chronic sterile inflammation drove and accelerated aging of hematopoietic stem cells and promoted aged hematopoietic phenotypes [88]. Naik et al. reported that skin epithelial stem cells possess the ability to remember prior inflammatory insults by maintaining long-term chromatin features acquired during the initial assault, enabling them to respond to subsequent injuries with accelerated proliferation and enhanced wound repair [94]. Similarly, in a mouse model of corneal epithelial injury, animals that had previously experienced and recovered from epithelial damage exhibited significantly faster epithelial regeneration when re-injured 28 days later [95]. These observations suggest that while tissue stem cells can adopt a primed, “locked-and-loaded” state to rapidly respond to repeated injury or inflammation, maintaining this heightened state chronically may ultimately lead to stem cell exhaustion.

## 6. Experimental Evidence Linking Ocular Surface Inflammation and Limbal Stem Cell Deficiency

### 6.1. In Vitro Studies

Studies conducted in laboratory settings have provided direct evidence of the damaging effects of inflammatory mediators on LSCs. For instance, research has demonstrated that key pro-inflammatory cytokines, such as IL-1β and TNF-α, which are typically elevated during ocular surface inflammation, can significantly reduce the ability of LSCs to proliferate and form colonies in culture condition [92,96]. This observation suggests a direct cytotoxic or inhibitory effect of these inflammatory molecules on the stem cell population. Furthermore, hyperosmotic stress, a condition often associated with inflammatory dry eye, has been shown to independently impair LSCs function by inducing cell death and causing a disruption in their normal cell cycle [7,97,98]. Notably, the combined presence of both pro-inflammatory cytokines and hyperosmotic stress has been found to have an even more severe negative impact on LSCs, indicating a synergistic damaging effect in the context of an inflamed and dry ocular surface.

Beyond specific cytokines, a broader inflammatory environment, such as that created by inflammatory and proangiogenic cytokines upregulated in response to stressors like ultraviolet (UV) radiation, has been shown to impair the stemness properties of LSCs and their ability to form colonies, suggesting a loss of their self-renewal capacity [99,100]. Culturing LSCs in the conditioned media from UV irradiated limbal fibroblasts induced the loss of stem cell markers [100]. Pro-inflammatory cytokines such as TNF-α and IFN-γ were significantly upregulated following cell irradiation of limbal fibroblasts. Interestingly, conditioned media derived from cultured ABCB5 LSCs stimulated the proliferation and migration of human blood and lymphatic endothelial cells in vitro [35]. This finding suggests a more complex role for LSCs, implying that they might not only be targets of inflammation but could also actively participate in the inflammatory and angiogenic processes observed in inflamed and LSCD-affected corneas.

### 6.2. In Vivo Studies

Animal models have been instrumental in demonstrating the link between ocular surface inflammation and the development of LSCD in a living organism. Studies in rabbits, for example, have shown that inducing partial or total LSCD leads to a significant increase in ocular surface inflammation, corneal neovascularization, and the ingrowth of conjunctival tissue onto the cornea [101,102]. The severity of these pathological changes was found to directly correlate with the degree of limbal damage, providing a clear cause-and-effect relationship between LSCD and the inflammatory response along with characteristic corneal changes [2,3]. Inflammation can disrupt and destroy the limbal niche and its surrounding microenvironment [103]. Research in mice has further highlighted the importance of the limbal niche in maintaining LSC function [104]. In animal models where LSCs were ablated, corneal epithelial cells demonstrated a capacity to migrate back to the limbus and transform into functional LSCs, suggesting a regenerative potential [3,105,106]. However, this regeneration was completely prevented when the limbal niche was experimentally destroyed, leading to conjunctivalization of the cornea. This finding underscores the critical role of the surrounding microenvironment in supporting LSC function and regeneration, and how inflammation that damages this niche can have severe consequences.

Another mouse study revealed that when LSCD was induced while preserving the structural integrity of the epithelial basement membrane, there was a less pronounced inflammatory response and a greater number of residual LSCs compared to when the basement membrane was also removed [107]. This suggests a protective role of the limbal microenvironment’s structural components against inflammatory damage to LSCs.

The ABCB5 knockout mouse model, which exhibits characteristics of LSCD, has also shown altered vascularization patterns in the limbus, indicating a role for LSCs in maintaining the normal avascularity of the cornea and suggesting that their deficiency can contribute to neovascularization in an inflammatory context [34,35]. Furthermore, studies using rodent models of alkali burns, a severe cause of ocular surface inflammation, have demonstrated a significant influx of inflammatory cells into the cornea, mediated by various signaling molecules like chemokines and adhesion molecules [108]. This intense inflammatory response can directly damage LSCs and their niche, ultimately leading to LSCD.

### 6.3. Clinical Findings: Association of Inflammatory Conditions with LSCD

Clinical observations have consistently demonstrated a strong link between chronic ocular surface inflammation and the development of LSCD (Table 7). Numerous conditions contribute to this inflammatory process, including dry eye disease, where chronic inflammation plays a key role in pathogenesis; blepharitis, which disrupts tear film stability; ocular rosacea, a chronic inflammatory condition affecting both skin and eyes; and allergic or atopic keratoconjunctivitis, marked by inflammatory responses to ocular allergens [7,109,110]. Severe immune-mediated disorders such as Stevens–Johnson syndrome (SJS), toxic epidermal necrolysis (TEN), mucous membrane pemphigoid (MMP), and graft-versus-host disease (GVHD) are also recognized for their association with LSCD [7,10,12,13,111,112]. Additionally, vernal keratoconjunctivitis (VKC) presents another allergic inflammatory contributor, while chronic limbitis directly affects the limbal region [10,12,13,111]. Other aggravating factors include inflammation and hypoxia from prolonged or improper contact lens wear, toxicity from preservative-containing topical medications, and ocular surface burns—whether chemical or thermal—that severely damage limbal stem cells. The broad spectrum of inflammatory conditions implicated in LSCD underscores chronic inflammation as a major risk factor for LSC damage and dysfunction in humans.

Recent study determined inflammatory cytokines in the tears of patients with unilateral total LSCD caused by chemical burns before and after autologous cultivated limbal epithelial stem cell transplantation (CLET) in 23 patients [113]. IL1β expression was significantly elevated in the LSCD eye compared with the unaffected eye preoperatively but decreased to normal level at 3 months after CLET. These findings suggest that LSCD itself exacerbates ocular surface inflammation, and that resolution of LSCD contributes to the control of this inflammation. In the study using the surgically harvested limbal pannus from eight total LSCD patients, conjunctivalization was accompanied by stromal vascularization and inflammatory cell infiltration in all specimens, supporting that inflammation plays a crucial role in LSCD [32].

**Table 7:** Specific Ocular Inflammatory Etiologies of LSCD and Their Characteristics.

Etiology	Summary
Dry Eye Syndrome (DES) and Meibomian Gland Dysfunction (MGD)	DES and MGD disrupt tear film homeostasis, leading to inflammation and damage to the central cornea and LSCs, resulting in LSCD. Treatment includes topical medications, blood products, amniotic membranes for DES, and meibomian gland expression, intense pulsed therapy, and probing for MGD [7].
Contact Lens (CL)-Induced LSCD	Contact lens-induced LSCD is characterized by whorl-like epitheliopathy and neovascularization. Pathogenesis involves tear film disruption, irritation from lens preservatives, and CL-induced inflammation, hypoxia, and hyperosmolarity. Treatment includes cessation of CL wear, topical steroids, artificial tears, and potentially surgical interventions like amniotic membrane transplant or limbal stem cell transplant [20].
Atopic and Vernal Keratoconjunctivitis (AKC/VKC)	AKC and VKC are allergic diseases causing conjunctiva edema, eyelid thickening, corneal scarring, neovascularization, and tear film instability. Inflammation damages the limbal niche, leading to LSCs loss. Treatment includes topical antihistamines, corticosteroids, immunomodulators, and systemic immunosuppression. Amniotic membrane transplantation with penetrating keratoplasty is useful in advanced cases [114].
Medication Toxicity-Induced LSCD	Medications like Mitomycin C, 5-fluorouracil, benzalkonium and systemic chemotherapy drugs (hydroxycarbamide, S-1) can cause LSCD. Treatment involves cessation of the medication, amniotic membrane transplantation, limbal transplantation and aggressive anti-inflammatory therapy [112,115].
Ocular Burn-Induced LSCD	Chemical or thermal burns cause corneal and limbal ischemia, leading to neovascularization and conjunctivalization. Severity is classified based on LSC loss. Treatment includes autologous serum, bandage contact lens, topical steroid and antibiotics and amniotic membrane or limbal stem cell transplantation [116].
Radiation-Induced LSCD	Radiation therapy can damage LSCs and results in corneal epithelial abnormality and inflammation leading to stem cell dysfunction and vision loss. Treatment includes topical steroids, artificial tears, autologous serum, and potentially surgical interventions like amniotic membrane transplant or limbal stem cell transplant in advanced LSCD [117].
Severe Infection-Induced LSCD	Severe ocular infections such as herpes simplex keratitis, herpes zoster ophthalmicus, microbial keratitis, and trachoma can lead to LSCD due to inflammation and damage to the limbal stem cells and niche. Infection control with adequate anti-microbial therapies is essential. Treatment includes cessation topical steroids, artificial tears, autologous serum, and potentially surgical interventions like amniotic membrane transplant or limbal stem cell transplant in advanced LSCD [118,119].
Stevens–Johnson Syndrome (SJS) and Toxic Epidermal Necrolysis (TEN)-Induced LSCD	SJS and TEN are severe immune reactions causing inflammation of the skin and mucous membranes leading to dry eye, corneal neovascularization, and LSCD. Management in the acute stage includes amniotic membrane grafting, systemic/topical corticosteroids, immunoglobulins, and cyclosporin A [120,121].
Graft vs. Host Disease (GVHD)	GVHD following hematopoietic stem cell transplantation can cause ocular surface inflammation and dry eye, potentially leading to LSCD. Treatment includes topical/systemic steroids, artificial tears, autologous serum, and potentially surgical interventions like amniotic membrane transplant or limbal stem cell transplant in advanced LSCD. Allogenic LSCs transplantation from the same bone marrow donor may be more favorable in long term management [122].
Pemphigoid-Related LSCD	Ocular mucous membrane pemphigoid is an autoimmune disease causing conjunctival scarring and potentially leading to corneal vascularization, opacification, and LSCD, possibly due to lacrimal duct scarring and severe dry eye. Treatment includes managing ocular surface disease, systemic immunosuppression, and preventing conjunctival fibrosis [123].
Rosacea-Induced LSCD	Ocular rosacea, a chronic inflammatory disease, can cause corneal neovascularization suggesting LSC damage. Treatment includes topical/systemic steroids, artificial tears, autologous serum, and potentially surgical interventions like amniotic membrane transplant or limbal stem cell transplant in advanced LSCD [110].
Bullous Keratopathy	Bullous keratopathy, characterized by a reduction in corneal endothelial cells, can lead to conjunctivalization of the peripheral cornea and delayed postoperative epithelialization, suggesting LSCD. Treatment includes penetrating keratoplasty or endothelial keratoplasty [124].
Ocular Surface Squamous Neoplasia (OSSN)	OSSN is a rare condition that can originate from the limbus and disrupt the Palisades of Vogt, potentially causing LSCD. Treatment includes excising malignancy, followed by limbal stem cell transplantation depending on the extent of the disease [125].

## 7. Underlying Mechanisms to Link Between Ocular Surface Inflammation and Limbal Stem Cell Deficiency:

The damage of LSC by ocular surface inflammation can occur through several interconnected pathways (Table 8). These multiple injurious stimuli exert synergistic effects, resulting in progressive and chronic damage to limbal stem cells.

### 7.1. Limbal Niche Destruction

One primary mechanism involves disruption of the limbal microenvironment, known as the limbal niche—a specialized structure crucial for maintaining LSC quiescence, self-renewal, and differentiation [56]. This niche consists of diverse cellular components, a complex extracellular matrix (ECM), and a finely regulated network of signaling molecules that sustain LSC function [56,57,61,126]. Persistent inflammation within the limbal stroma, a hallmark of LSCD, threatens the survival and efficacy of transplanted limbal epithelial stem cells [76]. Inflammatory and proangiogenic cytokines alter the niche’s gene expression and ECM protein profile, impairing LSC colony-forming capacity and stemness. Even in the absence of direct damage to LSCs, inflammation-induced alterations within the limbal niche can be detrimental. The specialized hyaluronan (HA) matrix within this niche plays a critical role in preserving LSCs in their stem cell state; however, chronic inflammation disrupts this essential component. Consequently, ongoing inflammation not only generates damaging cytokines but also undermines the structural and molecular integrity of the limbal niche, severely compromising LSC survival, self-renewal, and regenerative potential.

### 7.2. Inflammatory Cytokines and Cells

Inflammatory cytokines play a significant role in this process. For instance, cytokines like IL1β, TNF-α, and IL-8, which are elevated in conditions like dry eye, can directly induce inflammation in limbal epithelial cells [127,128] (Table 9). Notably, IL-1β has been found to be specifically elevated in the tears of patients with total LSCD. Exposure to agents like sulfur mustard, known to cause ocular injury, can also lead to delayed LSCD, accompanied by inflammatory cell infiltration in the limbus and cornea [129]. Even in the absence of direct injury to LSCs, chemical insults can trigger immune cell-mediated damage, involving changes in pH and the release of inflammatory cytokines within the anterior chamber and basal limbal tissue. In vitro studies also suggest that inflammatory signals such as TNF-α and IL-1β can stimulate the proliferation of various stem cell populations [130,131]. This phenomenon is likely applicable to early ocular surface inflammation as well. However, while such early inflammatory cues may initially play a beneficial role, their persistence in a chronic setting ultimately leads to stem cell exhaustion.

Persistent inflammation, regardless of the initial cause, appears to contribute to LSC dysfunction through multiple mechanisms. These include the continued secretion of pro-inflammatory cytokines, impairment of macrophage function in clearing debris, and the pathological stimulation of T-lymphocytes [132]. Furthermore, inflammation can lead to alterations within the limbal niche, affecting the extracellular matrix and adhesive molecules, ultimately resulting in the dysfunction of LSCs. Studies have also shown that the presence of inflammatory and proangiogenic cytokines can directly hamper the stemness and colony-forming efficiency of LSCs [7,97,133]. Recent mRNA sequencing of limbal epithelial cells from aniridia patients revealed that the top differentially expressed hub genes were predominantly associated with inflammatory and interferon-related pathways [134].

TNF-α inhibitor prevents LSCD and facilitated corneal reepithelialization after alkali burn [92,132]. Furthermore, inflammation can lead to alterations within the limbal niche, affecting the extracellular matrix and adhesive molecules, ultimately resulting in the dysfunction of LSCs. Studies have also shown that the presence of inflammatory and proangiogenic cytokines can directly hamper the stemness and colony-forming efficiency of LSCs [7,97,133]. Experimental dry eye model in rats activated p38 MAPK pathway, a key signal transduction pathway related to the production of pro-inflammatory cytokines, including IL-1β and TNF-α, and induced both limbal inflammation and LSC dysfunction. The inhibition of p38 MAPK pathway by specific inhibitor (SB203580) restored LSC proliferation [7].

### 7.3. Cornea Neovascularization

Corneal neovascularization is closely associated with ocular surface inflammation [25]. Inflammation of the ocular surface leads to an increase in various proangiogenic factors, which in turn induce neovascularization from the normal vascular network located in the limbus [135,136]. Among these factors, VEGFs, bFGF, and MMP-2 and MMP-9 play critical roles [137]. These molecules are secreted not only by damaged corneal cells but also by inflammatory cells, thereby creating a vicious cycle of inflammation and neovascularization.

Corneal neovascularization severely compromises LSC integrity by breaching the cornea’s avascular privilege and allowing inflammatory cells and cytokines to infiltrate the limbal zone [138,139]. This influx of immune components amplifies local inflammation and oxidative stress, triggering apoptosis and degradation of the LSC niche [138]. Altered oxygen and nutrient gradients due to aberrant vessel growth may impair LSC metabolic activity, while accompanying fibrosis and extracellular matrix remodeling physically can damage the palisades of Vogt, which house LSCs [58,140,141]. In addition, corneal nerves and neovascularization exhibit a mutually inhibitory relationship. Selective trigeminal denervation has been shown to trigger rapid-onset corneal neovascularization, whereas pharmacologically induced regional neovascularization, in turn, promotes local corneal denervation [142]. Altogether, corneal neovascularization transforms the limbal region into a hostile, inflamed, and structurally altered environment that erodes stem cell function and regenerative potential.

### 7.4. Cornea Nerve Damage

Ocular surface inflammation is closely related to cornea nerves and neurotrophic factors. NGF and other neurotrophic factors such as substance P are elevated in response to ocular surface inflammation. Experiment induction of ocular surface inflammation in rats using 0.1% benzalkonium chloride (BAK) reduced density of corneal subbasal nerve plexus, upregulated expression of substance P and inflammatory cytokines in both the cornea and trigeminal ganglion [143]. Elevated NGF was detected in tear film from active thyroid-associated ophthalmopathy patients [144]. Their role is to promote the healing process of cornea epithelium. NGF accelerates keratocyte migration, stimulates immunomodulation and healing after injury, induces epithelial cell proliferation and differentiation, and maintains LSCs [145,146]. Brain-derived neurotrophic factor (BDNF), and neurotrophin-3 (NT-3) also facilitate corneal wound healing in inflamed cornea [147].

However, corneal nerves can be damaged in the process of ocular surface inflammation. Corneal nerves are sensitive to immune-driven damage mediated by Th1 CD4+ T cells even in the absence of other pathogenic factors [148]. When chronic or severe inflammation leads to significant damage of corneal nerves, the beneficial roles of nerves in modulating ocular surface inflammation become impaired. This disruption can initiate a vicious cycle in which progressive nerve degeneration exacerbates inflammatory responses, further aggravating both inflammation and neural damage. As discussed, corneal nerve damage can induce cornea neovascularization and further damage ocular surface and limbal stem cell niche.

LSCD is intricately associated with corneal nerve dysfunction. Sensory nerves within the cornea supply essential neurotrophic factors—most notably NGF and substance P—that are critical for the survival, proliferation, and regenerative potential of LSCs [149]. Neural impairment leads to a reduction in these trophic signals, promoting stem cell apoptosis and impairing epithelial turnover. In the clinical study, sub-basal nerve density, length, and branch density were significantly reduced in LSCD eyes compared to controls [69].

In the clinical setting, Cenegermin^®^, a recombinant human nerve growth factor, improved clinical signs of LSCD [72]. Furthermore, nerve injury initiates sterile inflammatory cascades that mobilize immune cells, fostering the release of pro-inflammatory cytokines and reactive oxygen species (ROS) [150,151]. This inflammatory milieu aggravates damage to the limbal niche and accelerates disease progression. In parallel, deficient neurogenic feedback delays epithelial wound healing, predisposing the ocular surface to fibrotic remodeling and conjunctival trans-differentiation—pathological changes that permanently compromise the ocular surface [151].

### 7.5. Oxidative Damage of LSC

Ocular surface inflammation initiates a cascade of oxidative stress such as reactive oxygen species (ROS) that severely impact LSCs. Elevated levels of inflammatory cytokines—particularly TNF-α, IL-1β, and IFN-γ—activate ROS-producing enzymes like NADPH oxidase, leading to cellular damage through lipid peroxidation and DNA fragmentation [152]. Immune cell infiltration adds to this event; neutrophils and macrophages generate superoxide and hydrogen peroxide, which infiltrate the LSC microenvironment and induce mitochondrial dysfunction [153]. Chronic inflammation also weakens endogenous antioxidant defenses by downregulating enzymes such as superoxide dismutase, glutathione peroxidase, and catalase, tipping the redox balance toward persistent oxidative injury [154]. Environmental factors—like hyperosmolar tear film of hostile ocular surface, increased desiccation, and UV exposure—further intensify ROS production on the already compromised ocular surface [155]. Collectively, these insults degrade the stem cell niche significantly impairing the regenerative potential of LSCs.

## 8. Current Treatment Strategies and Management of Limbal Stem Cell Deficiency

### 8.1. Prevention and Early Intervention

Effective management of chronic ocular surface inflammation is paramount in mitigating the risk of developing limbal insufficiency [2,110]. Early recognition and appropriate treatment of underlying ocular surface conditions, such as allergic conjunctivitis, keratitis, and dry eye disease, are crucial preventative measures. Avoiding known triggers of ocular surface inflammation, including contact lens overwear and exposure to environmental irritants, can also help to minimize the risk of damage to the LSCs. Prompt and aggressive management of severe inflammatory conditions like Stevens–Johnson syndrome is critical to minimize the risk of long-term ocular complications, including LSCD [121]. Similarly, early diagnosis and initiation of systemic immunosuppression are vital in managing ocular cicatricial pemphigoid to halt disease progression and prevent subsequent LSCD [80]. Moreover, careful consideration should be given to the use of topical medications, especially those containing preservatives, to avoid inducing iatrogenic LSCD [115].

### 8.2. Medical Management

If the central 5 mm of the cornea is spared and the limbal involvement is within 6 h, medical management may be sufficient to promote improvement in LSCD. The goal of medical treatment is to prevent further damage and to restore the regenerative capacity of the corneal epithelium by preserving and stimulating LSCs through the recovery of the limbal niche. Currently, the most commonly employed medical interventions include ocular lubrication, eyelid management, and anti-inflammatory therapy [86,110].

The initial approach to managing LSCD often involves addressing any ongoing insults to the limbus [2]. This may include discontinuing the use of contact lenses, particularly if overwear or poor lens hygiene is suspected, or switching from topical medications containing preservatives to preservative-free formulations. A cornerstone of medical management is the frequent use of preservative-free artificial tears to provide lubrication and support the ocular surface. Topical corticosteroids are frequently prescribed to control inflammation, especially during acute exacerbations or in conditions with significant inflammatory components [156,157]. Immunomodulatory agents such as topical cyclosporine A and tacrolimus are also used to manage chronic inflammation and improve the health of the ocular surface [158]. Other medical treatments that may be considered include nightly application of topical Vitamin A ointment, and in cases of associated ocular rosacea, oral doxycycline [110]. Therapeutic contact lenses, including bandage contact lenses and scleral lenses, can provide corneal protection and, in some cases, improve vision [159,160]. In patients with significant aqueous tear deficiency, punctal occlusion, a procedure to block the tear drainage ducts, may be performed to prolong the effect of tear substitutes [110].

Autologous serum eye drops, enriched with EGF, Transforming Growth Factor-beta (TGF-β), fibronectin, vitamin A, and various cytokines, have been shown to restore a healthier ocular surface in patients with graft-versus-host disease, dry eye disease, Sjögren’s disease, and LSCD [161,162]. Similarly, platelet-derived preparations—including platelet releasate (PR), plasma rich in growth factors (PRGF), and platelet-rich plasma (PRP)—contain essential growth factors such as EGF, TGF, pigment-epithelium derived factor (PEDF), basic fibroblast growth factor (bFGF), and insulin-like growth factor-1 (IGF-1), which contribute to regenerating the limbal niche [163,164]. Additionally, amniotic membrane extract eye drops have demonstrated regenerative potential by facilitating the in vivo cultivation of limbal stem cells in LSCD patients [165,166]. This approach, along with other bioactive factors derived from amniotic membrane sources, presents promising strategies for enhancing LSC self-renewal and corneal epithelial healing.

Rho-associated protein kinase (ROCK) is a serine/threonine kinase that regulates cellular shape and adhesion through its action on the cytoskeleton [167,168]. ROCK inhibitors, widely used in glaucoma treatment, have recently shown promise in corneal disease therapy by enhancing cell proliferation, adhesion, and reducing apoptosis [169]. Currently, two commercially available ROCK inhibitors, Ripasudil and Netarsudil, are prescribed in ophthalmology as an eyedrop. Miyashita et al. demonstrated that culturing limbal epithelial cells (LECs) with keratinocyte growth factor and the ROCK inhibitor Y-27632 enhanced colony-forming efficiency and upregulated both progenitor/stem cell markers and epithelial markers [170]. Similarly, Sun et al. reported that Y-27632 increased colony formation, p63 and Ki67 expression, and the proportion of S-phase LECs [171]. Building on these findings, Kahuam et al. observed clinical resolution of LSCD following treatment with 0.4% ripasudil ophthalmic solution, highlighting the therapeutic potential of ROCK inhibition in LSCD management [172]. In addition, ROCK inhibitors can indirectly protect the limbal stem cell niche under ocular inflammatory conditions by suppressing corneal neovascularization. Zeng et al. and Sijnev et al. reported that ROCK inhibitor exhibited significant efficacy in inhibiting corneal neovascularization in animal models [173,174]. However, more robust evidence is still required to establish the efficacy of ROCK inhibitors in the management of LSCD.

### 8.3. Surgical Interventions

Limbal stem cell transplantation aims to replace damaged or deficient LSCs and is a key treatment for LSCD. Various techniques exist, including conjunctival limbal autograft (CLAU), where stem cells are transplanted from the healthy fellow eye; conjunctival limbal allograft (CLAL), using donor tissue from a living related or deceased donor; and ex vivo cultivated limbal epithelial cells (CLET), where a small biopsy of LSCs is expanded in a laboratory before transplantation. Simple limbal epithelial transplantation (SLET) is a newer, less invasive technique that utilizes a small amount of donor limbal tissue [175].

Amniotic membrane transplantation (AMT) can be used as an adjunctive procedure to promote healing and reduce inflammation. The basement membrane contains collagen Types IV and VII, fibronectin, laminin, as well as hyaluronic acid secreted by the epithelial layer [176]. Amniotic membrane (AM) provides multiple benefits for ocular surface reconstruction, including (a) anti-inflammatory effects (via hyaluronic acid and cytokine suppression), (b) anti-bacterial activity (via β-defenses and elafin), (c) anti-fibrotic properties (via TGF-β downregulation), (d) low antigenicity, and (e) immunomodulatory functions that prevent immune rejection [176,177]. While AM serves as an excellent biological scaffold for delivery of LSCs and provides a transient anti-inflammatory environment, its therapeutic utility remains significantly constrained in cases of total limbal niche destruction. The AM’s ECM—comprising collagens, laminin, and the specialized HC-HA/PTX3 complex—can temporarily mimic the native niche and promote LSC quiescence via BMP signaling [178]. The long-term success rate of amniotic membrane transplantation only in partial LSCD was reported as 40–54% at an average follow-up period of 52 months [179]. However, these benefits are often short-lived as the membrane typically degrades or is digested following transplantation, failing to provide a permanent structural foundation. Crucially, the reconstruction of a functional ocular surface in patients with severe LSCD requires more than just temporary cytokine delivery or a degradable scaffold. For patients whose limbal niche is completely obliterated, the AM cannot replicate or restore the complex, three-dimensional anatomical architecture essential for long-term stem cell maintenance. This includes the intricate network of sensory nerves, specialized vasculature, and the diverse population of limbal stromal cells that sustain the niche’s homeostasis [126]. Therefore, it must be acknowledged that while AM is a valuable tool for acute management and cell delivery, it cannot achieve the comprehensive anatomical and functional restoration of the limbus required for permanent clinical recovery in end-stage ocular surface disease.

In severe bilateral cases, a keratoprosthesis (artificial cornea) might be considered [180]. Addressing any underlying ocular surface comorbidities, such as dry eye or eyelid abnormalities, is also crucial for successful management [7].

## 9. Recent Advances in Diagnostic and Therapeutic Approaches of Limbal Stem Cell Deficiency

The field of LSCD continues to witness significant advancements in both diagnosis and treatment. Recent research has focused on refining diagnostic techniques and exploring novel therapeutic strategies to improve outcomes for patients suffering from this condition.

### 9.1. Emerging Diagnostic Approaches

In vivo confocal microscopy (IVCM) is a noninvasive, cellular-level imaging technique for the cornea, limbus, and conjunctiva [2,181]. It can detect both early and advanced features of LSCD including loss of palisades of Vogt, epithelial phenotype alteration, sub-basal nerve loss, neovascularization, and central epithelial thinning, all of which correlate with disease severity [182]. The presence of goblet cells on corneal IVCM is diagnostic of LSCD; however, their detection is limited by the small scan area, operator dependence, and variable reflectivity, often necessitating multiple-region scans to improve sensitivity [183]. LSCD severity is also associated with decreased basal epithelial cell density, increased cell size, nuclear hyperreflectivity, and a rise in stromal dendritic cells, which can aid in identifying partial LSCD [182]. Progressive degeneration of the corneal nerves is another hallmark, characterized by reduced sub-basal nerve density, shorter branch length, increased branching angles, and greater tortuosity [2,184].

Complementing these cellular-level findings, anterior segment optical coherence tomography (AS-OCT) is a repeatable, noninvasive tool for diagnosing LSCD by demonstrating epithelial and limbal thinning. LSCD typically shows 20–30% epithelial thinning versus ~10% in other epitheliopathies, making degree of thinning a useful discriminator. AS-OCT can visualize the palisades of Vogt and reveal marked epithelial thinning when they are absent. OCT angiography can image corneal and limbal vasculature and metrics like corneal vascular extension and thickness correlate with severity and vision [185].

Beyond imaging, molecular diagnostic approaches for LSCD continue to advance. Garcia et al. evaluated a reverse dot-blot PCR-strip assay for detecting MUC5AC mRNA in corneal epithelial samples from LSCD patients and reported a sensitivity of 98%, specificity of 89%, and positive and negative predictive values of 92% and 97%, respectively [186]. Liang et al. examined impression cytology specimens from healthy controls and LSCD patients using K12 and K13 immunostaining. All control samples demonstrated uniform K12 expression, whereas LSCD specimens showed a predominance of K13^+^ cells (93.8%) with only 2.6% exhibiting K12^+^/K13^+^ co-expression [187].

These diverse diagnostic modalities will undoubtedly contribute to faster and more accurate detection of LSCD, but their adoption in clinical practice is still hindered by high costs and the need for skilled operators experienced in image acquisition, especially for IVCM.

### 9.2. Recent Advances in Therapeutic Approaches

Recent advances in exosome biology and miRNA research are opening new avenues for LSCD therapy [188,189]. Several miRNAs—including miR-103, miR-107, miR-145, miR-10b, hsa-miR-143-3p, hsa-miR-10a-5p, hsa-miR-1910-5p, and hsa-miR-21-5p—are highly expressed in human LSCs [190]. Their predicted targets are enriched in pathways such as Wnt, PI3K–AKT, and MAPK, as well as networks regulating pluripotency, migration, growth, and proliferation [190]. Exogenous delivery of these miRNAs may therefore enhance LSC populations and offer therapeutic benefit in LSCD. Emerging evidence shows that miR-146a and miR-10b are differentially expressed in exosomes derived from diabetic versus healthy limbal progenitor cells, suggesting a role in LSC homeostasis [191]. Inhibition of miR-146a improved epithelial healing in diabetic human organ-cultured corneas, highlighting its therapeutic potential [192]. While miRNA-based strategies are promising, several limitations must be carefully considered before clinical translation [193]. Effective delivery remains a major challenge due to ocular surface barriers, tear dilution, rapid clearance, and limited stromal penetration [194]. miRNAs are also highly susceptible to enzymatic degradation, often requiring specialized carriers to enhance stability and cellular uptake [194]. In addition, off-target effects pose a significant concern, as a single miRNA can modulate hundreds of genes across multiple signaling pathways, potentially disrupting normal cellular functions, immune responses, tissue homeostasis, and repair mechanisms [195]. The possible consequences of prolonged or excessive modulation of miRNA activity should also be addressed, as sustained dysregulation may lead to unintended biological effects.

In parallel, exosomes derived from mesenchymal stem cells (MSCs) have demonstrated pro-regenerative effects. Samiel et al. reported that MSC-derived exosomes enhanced LSC growth by activating Wnt/β-catenin, p38 MAPK, and ERK signaling pathways [196], while Li et al. showed that adipose-derived MSC exosomes promoted LSC colony-forming capacity [197]. Although exosome-based therapies are promising, several obstacles continue to limit their clinical translation. Key challenges include inconsistent manufacturing, limited scalability, safety concerns, low delivery efficiency, poor ocular retention, and high production costs [198]. Topical administration further complicates delivery, as exosomes must overcome rapid tear turnover, the negatively charged and lipophilic corneal epithelium, and the hydrophilic stroma—barriers that collectively restrict penetration and diminish therapeutic efficacy [198]. Additional limitations arise from the intrinsic properties of natural exosomes, including their lack of specific targeting capability, substantial heterogeneity, and the complexity of their therapeutic mechanisms and cargo composition [199].

Beyond exosome-based approaches, cell-based therapies continue to evolve. Cultivated autologous limbal epithelial cell (CALEC) transplantation has emerged as a promising option, with clinical trials demonstrating feasibility, safety, and encouraging early outcomes in patients with severe LSCD [43]. Induced pluripotent stem cell (iPSC)-derived corneal epithelial cells are also being explored, with initial studies reporting successful restoration of corneal surface integrity and visual improvement [200]. These efforts align with broader research aimed at understanding and regenerating the limbal stem cell niche, including the roles of niche-derived factors and signaling pathways.

As discussed, MSC-derived exosomes, in particular, have been shown to enhance LSC proliferation [196], and MSCs themselves can modulate inflammation and secrete trophic factors that support niche reconstruction [201,202]. In animal models of alkali burn, topical or subconjunctival administration of limbal MSCs reduced corneal opacity, attenuated neovascularization, and improved epithelial healing [203].

In addition to LSC-based strategies, evidence suggests that progenitor-like cells exist within the central cornea and may contribute to epithelial regeneration [44,105,204,205]. LSCs typically divide asymmetrically to generate one LSC and one TAC. Because TACs represent an intermediate state between stem and differentiated cells, they may correspond to the progenitor-like population observed in the cornea. Their high proliferative potential and capacity for population expansion make them attractive candidates for regenerative approaches. Studies in other tissues have shown that stem cell pools can be replenished through dedifferentiation of mature cells [206,207], and similar phenomena have been observed on the ocular surface. In mouse models, putative LSC markers have been detected in both the limbus and cornea, and after complete limbal debridement, peripheral corneal epithelial cells migrated centrifugally to repopulate the limbus and re-express LSC markers [105,106]. However, TACs have a limited lifespan compared with true stem cells, raising the risk of long-term corneal surface exhaustion in the absence of a stable LSC source.

Building on this concept, differentiated corneal epithelial cells—readily obtainable from patients—are being investigated as a source for generating LSC-like cells through induced dedifferentiation. Human limbal epithelial cells have been successfully reprogrammed into iPSCs and subsequently re-differentiated into limbal-like epithelial cells expressing characteristic LSC markers, demonstrating potential for corneal surface regeneration [208]. Because dedifferentiation is strongly influenced by the limbal niche, strategies that recreate or enhance niche conditions may enable TAC- or epithelial-cell-derived LSC generation. These approaches may ultimately provide an effective strategy to stabilize or even reverse LSCD progression, potentially delaying or obviating the need for surgical intervention [208]. However, before clinical application, significant concerns remain, including tumorigenicity risks, complex differentiation protocols, and stringent regulatory hurdles [209]. In addition, advanced cultured methods require expensive Good Manufacturing Practice (GMP) facilities, limiting widespread accessibility. Furthermore, clinical outcomes remain highly variable, and the optimal culture, surgical, and medical protocols have not yet been globally standardized. Finally, the exact long-term fate and therapeutic mechanisms of transplanted stem cells on the ocular surface remain uncertain [210].

Gene-based therapies are also emerging as potential treatments. For example, Yang et al. used AAV-mediated delivery of FOXC1 in PAX6+/− mice, a model of LSCD, and observed increased expression of LSC markers such as N-cadherin and Lrig1 [211]. Gene therapy offers the potential for long-lasting tissue repair by targeting specific molecular pathways; however, its clinical application in ocular surface diseases remains constrained by several biological and safety challenges. Major concerns include off-target effects, potential insertional mutagenesis, and immune reactions to viral vectors commonly used for gene delivery. Efficient delivery is further impeded by ocular physiological barriers that limit vector penetration, including rapid tear washout, blinking, and the tight junctions of the corneal epithelium. The rapid turnover of corneal epithelial cells also results in transient gene expression, necessitating repeated administrations to sustain therapeutic benefit. Although viral vectors provide superior transfection efficiency, they carry risks of immunogenicity and ocular surface inflammation [212]. Conversely, non-viral vectors offer improved safety profiles but exhibit low transfection efficiency, highlighting the need for continued advances in nanotechnology-based carriers and targeted delivery systems [213].

## 10. Therapeutic Potential of Selectively Targeting Inflammation in Limbal Stem Cell Deficiency

As the link between ocular surface inflammation and LSCD has become increasingly important, recent studies have actively explored inflammation-targeted approaches for the treatment of LSCD.

High-density lipoproteins (HDLs) play an important anti-inflammatory role on the ocular surface by targeting epithelial and innate immune cells to suppress key inflammatory pathways and promote resolution [214]. Building on this concept, a recent study demonstrated that HDL-mimetic nanoparticles containing pentaerythritol tetraoleate (PE-O_4_) and pentaerythritol tetrastearate (PE-S_4_) significantly reduced corneal haze, attenuated inflammation, and suppressed conjunctivalization and corneal neovascularization in a nitrogen-mustard keratopathy mouse model [215]. These findings highlight the therapeutic potential of lipid-based nanocarriers in restoring ocular surface homeostasis.

Environmental insults to the cornea are well-known activators of NF-κB, a central regulator of inflammation, cell proliferation, and survival. Inhibition of NF-κB signaling has been shown to prevent corneal haze, neovascularization, and impaired epithelial regeneration [216,217,218,219,220]. Because NF-κB acts as a master switch for inflammatory cytokines that disrupt the limbal niche, targeted modulation of this pathway represents a promising strategy for preserving LSC stemness. Current approaches include HC-HA/PTX3 complexes derived from amniotic membranes, which sequester pro-inflammatory mediators and block NF-κB nuclear translocation [221]. Pharmacologic agents such as IKK inhibitors and localized corticosteroids stabilize IκB to keep NF-κB inactive, while antioxidants like N-acetylcysteine (NAC) reduce ROS-mediated activation [222]. Emerging regenerative therapies using MSC-derived exosomes further enhance this strategy by delivering microRNAs that silence NF-κB-related genes [223]. Although systemic NF-κB inhibition may compromise immune surveillance, localized ocular surface modulation—via biomimetic scaffolds or secretome-based therapies—offers a targeted means to suppress chronic inflammation, prevent aberrant differentiation, and maintain a supportive microenvironment for LSC survival [216,217,218,219,220,224].

Another inflammatory pathway relevant to LSCD involves mineralocorticoid receptor (MR) overactivation, which promotes oxidative stress, inflammation, fibrosis, and neuropathy [225,226]. MR antagonists have shown therapeutic benefit across ocular disease models: intravitreal administration reduced retinal edema and inflammation in diabetic retinopathy, while systemic and local delivery suppressed choroidal neovascularization through VEGF-independent mechanisms [227,228]. In the context of LSCD, spironolactone and eplerenone reduced corneal neovascularization and edema, with spironolactone additionally limiting inflammation, strengthening the epithelial barrier, reducing conjunctivalization, and promoting nerve regeneration [229]. Topical spironolactone also improved signs of ocular GVHD, including epithelial damage, meibomian gland loss, and inflammatory infiltration [230].

Pro-inflammatory cytokines such as TNF-α and IL-1β are major drivers of the “cytokine storm” following ocular surface injury and play a central role in destabilizing the limbal niche [231]. These cytokines degrade extracellular matrix components, activate NF-κB, and induce LSC apoptosis. Targeted biologic therapies have shown promise in counteracting these effects. Systemic infliximab, a TNF-α inhibitor, prevented LSC deficiency and accelerated corneal re-epithelialization after severe alkali burns by neutralizing local inflammation [92]. Similarly, blockade of IL-1β signaling reduced pathological neovascularization and inhibited conjunctivalization of the cornea [232,233]. By stabilizing the biochemical environment of the limbus and preventing LSC loss, cytokine-specific biologics offer a more precise and effective alternative to broad immunosuppression.

## 11. Conclusions

The relationship between ocular surface inflammation and LSCD is deeply interconnected, as chronic or severe inflammation directly undermines the viability of LSCs and disrupts the integrity of their specialized niche. Because nearly all predisposing risk factors for LSCD involve some degree of inflammatory activity, a thorough understanding of the shared molecular and cellular mechanisms driving this interaction is essential for accurate diagnosis and effective clinical management. Although ambiguity surrounding definitive LSC markers remains an unresolved challenge, this may indicate that LSCs are not a uniform cell population defined by a single characteristic, but rather a dynamic and complex group of cells that express different markers depending on their microenvironment. Advances in ophthalmic imaging and molecular diagnostic technologies have also enabled earlier and more accurate detection of LSCD than ever before, expanding treatment opportunities for patients.

Current treatment strategies span a broad continuum, from intensive medical therapies aimed at suppressing inflammation and stabilizing the ocular surface to sophisticated surgical procedures and emerging cell-based transplantation techniques designed to restore the limbal stem cell population. When initiated early, aggressive control of inflammation can significantly slow or even halt disease progression. Despite the promise of regenerative approaches, many experimental diagnostic and therapeutic modalities still face substantial barriers, including high costs and stringent regulatory requirements.

The future of LSCD management will depend on integrating established clinical expertise with cutting-edge biotechnological tools to clarify the precise pathways through which inflammation induces LSC damage. By focusing research on these fundamental mechanisms and developing targeted strategies to enhance the regenerative capacity of residual LSCs, the field can better mitigate this vision-threatening condition and improve long-term outcomes for affected patients.

## Figures and Tables

**Table 1:** Causes of Ocular Surface Inflammation.

Category	Specific Examples
Systemic Disorders	Graft versus host disease, Stevens Johnson syndrome, cicatrizing conjunctivitis
Environmental Factors	Dry air, wind, dust, microparticles, smoke, fume, allergens (dust, pollen, and grass)
Infections	Bacterial keratoconjunctivitis, viral keratitis, fungal keratitis
Allergies	Allergic conjunctivitis (seasonal, perennial, vernal, and atopic)
Autoimmune Diseases	Sjögren’s syndrome, ocular rosacea, mucous membrane pemphigoid
Trauma/Injury	Chemical burns, thermal burns, ocular surgery
Medications	Antihistamines, antidepressants, anticancer drug, topical glaucoma medications (especially with preservatives)
Contact Lens Wear	Poor fitting and hygiene, extended wear, sensitivity to solutions, contamination
Meibomian Gland Dysfunction	Terminal duct obstruction, qualitative/quantitative changes in lipid secretion
Other Ocular Conditions	Blepharitis, uveitis, episcleritis, scleritis, superficial punctate keratitis, neurotrophic keratitis

**Table 3:** Cues for TAC proliferation after epithelial injury.

Key Molecular Signaling Pathways	
ACE2-TGFα-EGFR-LCN2 Axis	Corneal injury causes a downregulation of ACE2, which in turn activates limbal epithelial and TACs proliferation. This occurs via a pathway involving TGFα and EGFR [48]
EGFR Transactivation	EGFR acts as a central mediator, converging multiple extracellular “injury” signals into intracellular pathways like PI3K/Akt and ERK pathways [49]
ATP and “Alarmins”	Injured cells release extracellular ATP, which acts as a “damage signal” to activate P2Y receptors, leading to EGFR transactivation [50]
Soluble Growth Factors and Cytokines	
EGF and Heparin binding EGF (HB-EGF)	Potent mitogens that initiate DNA synthesis and cell division [50]
KGF and HGF	Potent mitogens that initiate DNA synthesis and cell division. KGF enhances proliferation of TACs while HGF enhances both proliferation and motility of TACs [50]
CNTF	Upregulated after injury and directly promotes the expansion of progenitor-like cells [51]
Physical and Environmental Cues	
Loss of Contact Inhibition	The physical defect in the epithelium reduces spatial constraints, signaling neighboring cells to enter the cell cycle and migrate into the wound bed [52]
Extracellular Matrix (ECM) Changes	Wounding alters the basement membrane, exposing TACs to cues like Fibronectin and Laminin, which guide their migration and support proliferation [52]
Nerve Damage	Corneal nerve injury itself can disrupt normal homeostasis, inducing a marked and sustained increase in epithelial proliferation to compensate for the loss of neurotrophic support [53]
Immune Cell Contribution	
Neutrophils and T-cells	Infiltrating neutrophils and γδ T-cells release growth factors (like IL-22) that directly stimulate corneal epithelial division and migration [54]
Amphiregulin	Resident innate lymphoid cells in the limbus produce amphiregulin, an EGF-like factor, to drive rapid cell division following injury [55]

**Table 4:** Components of the limbal stem cell niche and their roles in LSC maintenance.

Category	Components	Role in LSC Maintenance
Cellular	Stromal cells, melanocytes, immune cells	Paracrine support, protection
ECM	HA, basement membrane	Stemness preservation
Growth factors	EGF, NGF, HGF, IGF-1, PEDF	Proliferation, survival
Signaling	Wnt, Notch, BMP, Shh	Fate regulation
Neural	NGF, CNTF	Colony-forming efficiency

**Table 5:** Stages of limbal stem cell deficiency.

Functional Stage	Definition	Stage A	Stage B	Stage C
Stage 1	Central 5 mm corneal epithelium preserved	Stage 1A Early LSCD with preserved visual axis and substantial residual limbal stem cell reservoir	Stage 1B Preserved central vision with significant limbal stem cell loss	Stage 1C Preserved central epithelium despite complete circumferential limbal deficiency
Stage 2	Central 5 mm corneal epithelium damaged	Stage 2A Visual axis involvement with residual limbal stem cells	Stage 2B Visual axis involvement with marked reduction in limbal stem cells	Stage 2C Visual axis involvement with near-complete loss of limbal stem cells
Stage 3	Entire corneal epithelium compromised	Stage 3A Severe LSCD with limited residual limbal stem cells	Stage 3B Severe LSCD with minimal limbal stem cell reserve	Stage 3C End-stage LSCD with complete loss of functional limbal stem cell niche

**Table 6:** Distinction between limbal stem cell depletion and dysfunction.

	LSC Depletion	LSC Dysfunction
Stem cell status	Actual loss of LSC population	Residual LSCs preserved but functionally impaired
Primary mechanism	Severe acute injury or long-standing chronic injury	Chronic inflammation, genetic defects, environmental stress
Reversibility	Largely irreversible	Potentially reversible
Conjunctivalization pattern	Diffuse, 360° involvement	Patchy or peripheral involvement
Central cornea	Loss of corneal epithelial phenotype	CK12 or K3 expressions are often preserved
Impression cytology	CK12-, Muc5ac+	CK12+ centrally
Therapeutic focus	Stem cell replacement	Niche restoration and inflammation control
Clinical implication	Requires transplantation	Early intervention may prevent progression

**Table 8:** Inflammation-driven mechanisms leading to limbal stem cell dysfunction and depletion.

Mechanism	Key Mediators/Events	Impact on LSCs
Niche destruction	HA loss, ECM remodeling	Stemness ↓
Cytokine toxicity	IL-1b, TNF-a	Proliferation, apoptosis ↓
Neovascularization	VEGF, MMPs	Barrier breakdown
Nerve damage	NGF loss	Trophic support ↓
Oxidative stress	ROS	DNA/mitochondrial damage

**Table 9:** Key Inflammatory Mediators and Their Role in Limbal Stem Cell Dysfunction.

Inflammatory Mediator	Primary Role in Inflammation and Potential Impact on LSCs/LSCD
IL-1β	Potent pro-inflammatory; can inhibit LSC proliferation and induce apoptosis.
TNF-α	Pro-inflammatory; can inhibit LSC proliferation and survival.
IL-6	Pro-inflammatory; stimulates inflammatory cells and VEGF secretion, contributing to neovascularization.
IL-8	Attracts neutrophils; can induce corneal neovascularization.
MCP-1/CCL2	Recruit monocytes and macrophages, contributing to chronic inflammation.
VEGF	Potent pro-angiogenic factor; upregulated in inflamed corneas with LSCD, leading to neovascularization. IL-6 can stimulate its production.
MMPs	Involved in ECM degradation and tissue remodeling during inflammation; can contribute to corneal damage and neovascularization.
Adhesion molecules	Facilitate the recruitment of inflammatory cells to the limbal region and cornea.
Reactive oxygen species	Can cause oxidative damage to LSCs and the limbal microenvironment.

## Data Availability

No new data were created or analyzed in this study. Data sharing is not applicable to this article.
